# Mitonuclear Mismatch is Associated With Increased Male Frequency, Outcrossing, and Male Sperm Size in Experimentally-Evolved *C. elegans*

**DOI:** 10.3389/fgene.2022.742272

**Published:** 2022-03-11

**Authors:** Brent W. Bever, Zachary P. Dietz, Jennifer A. Sullins, Ariana M. Montoya, Ulfar Bergthorsson, Vaishali Katju, Suzanne Estes

**Affiliations:** ^1^ Department of Biology, Portland State University, Portland, OR, United States; ^2^ Department of Veterinary Integrative Biosciences, Texas A&M University, College Station, TX, United States

**Keywords:** adaptation, compensatory evolution, electron transport chain, mating behavior, mitochondria, selfing, sexual conflict, sperm size

## Abstract

We provide a partial test of the mitonuclear sex hypothesis with the first controlled study of how male frequencies and rates of outcrossing evolve in response to mitonuclear mismatch by allowing replicate lineages of *C. elegans* nematodes containing either mitochondrial or nuclear mutations of electron transport chain (ETC) genes to evolve under three sexual systems: facultatively outcrossing (wildtype), obligately selfing, and obligately outcrossing. Among facultatively outcrossing lines, we found evolution of increased male frequency in at least one replicate line of all four ETC mutant backgrounds tested—nuclear *isp-1*, mitochondrial *cox-1* and *ctb-1*, and an *isp-1 IV; ctb-1M* mitonuclear double mutant—and confirmed for a single line set (*cox-1*) that increased male frequency also resulted in successful outcrossing. We previously found the same result for lines evolved from another nuclear ETC mutant, *gas-1*. For several lines in the current experiment, however, male frequency declined to wildtype levels (near 0%) in later generations. Male frequency did not change in lines evolved from a wildtype control strain. Additional phenotypic assays of lines evolved from the mitochondrial *cox-1* mutant indicated that evolution of high male frequency was accompanied by evolution of increased male sperm size and mating success with tester females, but that it did not translate into increased mating success with coevolved hermaphrodites. Rather, hermaphrodites’ self-crossed reproductive fitness increased, consistent with sexually antagonistic coevolution. In accordance with evolutionary theory, males and sexual outcrossing may be most beneficial to populations evolving from a state of low ancestral fitness (*gas-1*, as previously reported) and less beneficial or deleterious to those evolving from a state of higher ancestral fitness (*cox-1*). In support of this idea, the obligately outcrossing *fog-2 V; cox-1 M* lines exhibited no fitness evolution compared to their ancestor, while facultatively outcrossing lines showed slight upward evolution of fitness, and all but one of the obligately selfing *xol-1 X; cox-1 M* lines evolved substantially increased fitness—even beyond wildtype levels. This work provides a foundation to directly test the effect of reproductive mode on the evolutionary dynamics of mitonuclear genomes, as well as whether compensatory mutations (nuclear or mitochondrial) can rescue populations from mitochondrial dysfunction.

## Introduction

Despite decades of research effort, the reasons why sex evolved and why it is so widely maintained despite its costs remain incompletely understood (Smith, 1978; Sharp and Otto, 2016). One of the major classes of hypotheses to explain the evolution and persistence of sex centers on the problem of selective interference among loci—the Hill-Robertson effect, which describes the reduced efficiency of selection that occurs at one site due to selection at a linked site (Felsenstein and Yokoyama, 1974; Otto, 2021). By disassociating beneficial from deleterious mutations, for example, recombination and outcrossing can enable faster adaptation to new conditions and purge more deleterious mutations from nuclear genomes than selfing or asexuality (Otto and Lenormand 2002; Whitlock 2002; Marchini et al., 2016). Laboratory studies such as those utilizing *Caenorhabditis elegans* nematodes have consistently shown large short-term benefits of outcrossing in populations adapting to a new challenge, but that outcrossing rates often plummet after a few generations (e.g., Morran et al., 2009a , 2009b; reviewed in; Cutter et al., 2019). Once outcrossing populations have utilized their standing genetic variation to attain the most fit genotype, further outcrossing would only serve to break apart any beneficial linkage arrangements it had previously helped to achieve, and selfing will again be favored in keeping with the reduction principle (Otto and Lenormand, 2002). Thus, sustained or dynamic selection is presumably required to maintain outcrossing. The agents of such sustained selection are often considered to be deleterious mutations or coevolving parasites. The Red Queen hypothesis suggests that host-parasite coevolution (or other types of temporal environmental change) causes persistent negative frequency-dependent selection that favors outcrossing (Jaenike, 1978; Hamilton, 1980). Parasites will infect the most common genotype and, because outcrossing produces a greater diversity of genotypes, sexually reproducing lineages are expected to better survive under this parasitic pressure (e.g., Koskella and Lively, 2009; Morran et al., 2011, 2012). Recently, the “mitonuclear sex hypothesis”, has been advanced to explain the evolution and maintenance of sex (Havird et al., 2015). Described by the authors as a “Red Queen hypothesis with a mitonuclear twist”, it is the first to consider a potential role for mitochondrial-nuclear genetic interactions in the early emergence and maintenance of sexual recombination in eukaryote evolution. The current study seeks to provide an initial test of this hypothesis using *C. elegans* nematodes as a model.

Maintenance of favorable epistasis between mitochondrial and nuclear genomes is critical to proper mitochondrial function and therefore to the maintenance of nearly all eukaryotic life (Dowling et al., 2008). For example, the electron transport chain (ETC) that operates within the mitochondrial inner membrane to synthesize ATP relies upon both mitochondrial DNA (mtDNA) and nuclear DNA (nDNA) encoded proteins and RNAs to function (Burton et al., 2013). The coevolution between nDNA- and mtDNA-encoded subunits of the ETC is demonstrated by positively correlated rates of gene and protein sequence evolution (Barreto et al., 2018) as well as reduced fitness of hybrid lineages with incompatible mitonuclear combinations (Rawson and Burton, 2002). Such mitonuclear coadaptations have been hypothesized to be primarily maintained by nuclear-genome driven compensatory coevolution (Burton et al., 2013; Hill, 2020, but see; Piccinini et al., 2021). This hypothesis posits that mitonuclear matching is maintained by nDNA mutations that serve to compensate for ongoing mitochondrial genome decay believed to result from features of mitochondrial population biology (e.g., maternal inheritance, lack of recombination, and limited DNA repair; Ballard and Whitlock, 2004).

A direct extension of the nuclear compensation hypothesis, the mitonuclear sex hypothesis argues that mutation pressure by the endosymbiotic mitochondrial ancestor was a major driver of sexual outcrossing in the host, and that sex is maintained to increase the frequency of new combinations of host nDNA alleles available to compensate for the hypothesized, ongoing mtDNA decay and associated deleterious metabolic effects (Havird et al., 2015). These effects could have resulted from internal reactive oxygen species (ROS) generated by the bacterial endosymbiont, i.e., pre-mitochondrion (Hörandl and Speijer, 2018). The benefits of the mitonuclear sex hypothesis would not be extended to asexually transmitted mtDNA. This hypothesis further predicts that, in facultatively outcrossing lineages, outcrossing will be favored over asexuality in cases of mitonuclear mismatch. Whether such mtDNA genome deterioration is occurring is an ongoing topic of debate, and a recent molecular population genetic study in bivalves finds evidence for mitonuclear coevolution in the absence of nuclear compensation (Piccinini et al., 2021). The mitonuclear sex hypothesis also fails to adequately explain why asexual reproduction arises and persists in many groups that should experience mtDNA decay across the tree of life. However, there is an almost complete lack of data, and no controlled studies of the effects of reproductive mode on mitonuclear evolutionary dynamics have been done. Furthermore, how the rate of outcrossing affects the rate and dynamics of evolution in direct response to mitonuclear mismatch has not been experimentally studied.

Importantly for the current study, *C. elegans* is androdiecious with populations normally comprising self-fertilizing hermaphrodites (XX) and extremely rare males (hemizygous, XO) with whom hermaphrodites can outcross. Hermaphrodites produce sperm during larval development, then switch to producing oocytes that are subsequently fertilized by their own sperm (Kuwabara and Kimble, 1992). Males are generated by nondisjunction of the X chromosome, which occurs spontaneously at a rate of 1/1,000 in the standard Bristol N2 laboratory strain, but occasionally at higher rates within some natural isolates (Teotónio et al., 2006). Rates of chromosome non-disjunction, and thus frequencies of *C. elegans* males, can be experimentally increased by exposing developing hermaphrodites to stressors such as heat (Sulston and Hodgkin, 1988) or ethanol. Because larger male sperm outcompete smaller hermaphrodite self sperm (LaMunyon and Ward, 1997), outcrossed offspring exhibit 50:50 sex ratios, but males are then rapidly lost from populations under most circumstances (Stewart and Phillips, 2002; Teotonio et al., 2006). This occurs despite the fact that hermaphrodites are sperm-limited and can substantially increase their reproductive output by outcrossing with males. This loss of males appears to be ultimately due to hermaphrodite mating avoidance (reviewed in Cutter et al., 2019). However, laboratory *C. elegans* populations exposed to selection can evolve and/or maintain males at high frequencies in the absence of increased X chromosome nondisjunction (Morran et al., 2009b; Anderson et al., 2010; Teotonio et al., 2012), consistent with recombination and outcrossing being favored to facilitate adaptive evolution. *Caenorhabditis elegans* is unique among metazoan experimental systems in that the mating system can be genetically manipulated. Populations with variable ratios of males, females, and hermaphrodites—and thus different degrees of selfing and outcrossing—can be obtained; this allows direct tests of the role of outcrossing in evolution (Katju et al., 2018) and conversely, of how rates of outcrossing can evolve in response to various treatments. Such manipulations have revealed the large extent to which *C. elegans’* mating system is shaped by the avoidance of sexual conflict (reviewed in Cutter et al., 2019). For instance, experimental evolution of mixed populations of *C. elegans* under conditions of obligate outcrossing resulted in rapid evolution of larger and more competitive male sperm and male mating traits that harmed their mates (LaMunyon and Ward, 1997; Palopoli et al., 2015). Finally, *C. elegans* harbor no *Wolbachia* bacteria or other sex-ratio distorting intracellular parasites, nor do they experience inbreeding depression, and their mtDNA exhibits no evidence of paternal leakage (Kang et al., 2016) or frequent recombination (Burton et al., 2013)–all features that simplify interpretation of experimental outcomes. *Caenorhabditis elegans*’ amenability to laboratory experimental evolution (reviewed in Gray and Cutter, 2014; Teotonio et al., 2017) and the availability of several well-characterized nDNA- and mtDNA-encoded mutants affecting mitochondrial function (Van Der Bliek et al., 2017) make it ideal for conducting experimental evolution to understand the impact of mitonuclear mismatch on the evolution of outcrossing.

In this study, we draw from one finding of our previous experiment (Wernick et al., 2019), which found that lineages of *C. elegans* nematodes evolving from a state of extreme mitonuclear mismatch in the form of a function-disrupting ETC mutation tended to achieve greater fitness gains when they also evolved high male frequency (and presumably, outcrossing). Although high male frequency was not a prerequisite for adaptation, the six (of 24) lines that evolved high male numbers showed a strong tendency for greater adaptation (Figure 1 in Wernick et al., 2019). These results were consistent with sex being favored over the course of the 60-generation experiment in cases of mitochondrial mismatch, and despite approximately zero starting genetic variation in the ancestral population. Here we apply the same experimental evolution procedure with an array of mitonuclear mismatched strains and a wildtype control strain, each one evolving within three sexual systems—facultatively outcrossing (wildtype), obligately outcrossing, and obligately self-fertilizing—in order to test the mitonuclear sex hypothesis and evaluate biological changes associated with altered rates of outcrossing. Because our study does not include asexual lineages, but rather selfing lineages wherein a low level of effective recombination between mitochondrial and nuclear types (i.e., new combinations of mitochondrial and nuclear genomes) will occur, we are unable to provide a perfect test of this hypothesis. Our results nonetheless suggest that increased male frequency and outcrossing may be favored by facultatively outcrossing lineages experiencing mismatched mitonuclear genomes. We report evolution of male frequency for all strains, and then provide a detailed phenotypic analysis of a single group of lines—those containing the mitochondrial *cox-1* mutation. Phenotypic analyses of these lines indicate that evolution of high male frequency was accompanied by increased male sperm size and changes in male mating success indicative of sexually antagonistic coevolution. We additionally report relative population fitness for the same *cox-1*-containing lines and interpret the results in the context of evolutionary theory. This study is still ongoing and full phenotypic and whole-genome analyses for all ETC mutant lineages will be reported in future manuscripts.

## Methods

*Strains.* Strains containing mutations in ETC genes listed in Table 1 were generated on Katju laboratory wild-type N2 (facultatively outcrossing), and on *xol-1* (selfing) and *fog*-2 (outcrossing) deletion-mutant genetic backgrounds. The *xol-1* and *fog-2* deletion alleles were generated *via* CRISPR-Cas9 technology (NemaMetrix/InVivo Biosystems, Eugene, OR). Sperm production in hermaphrodites is initiated by the FOG-2 protein; disruption of the *fog-2* gene leads to feminization of the hermaphrodites and thus obligately outcrossing populations consisting of equal proportions of functional females and males (Clifford et al., 2000). XOL-1 is the master sex-determination switch gene that functions in X-chromosome dosage compensation; disruption of this function in *xol-1* mutants causes male (XO) lethality and results in obligate selfing (Miller et al., 1988). The nuclear-encoded ETC mutant alleles, *gas-1* and *isp-1*, were also generated by CRISPR-Cas9 editing to create deletion knockout mutations. Conversely, the mitochondrial-encoded ETC mutant alleles, *ctb-1* and *cox-1*, are single base-pair substitutions that were integrated onto the N2 nuclear background *via* 10 rounds of backcrossing of *ctb-1* or *cox-1* hermaphrodites with N2 males. The *ctb-1*(*qm189*) mutant was obtained from the Caenorhabditis Genetics Center (CGC; University of Minnesota); the *cox-1* mutant was kindly provided by Dr. Marni Falk (Children’s Hospital of Philadelphia). Finally, we utilized a previously characterized *isp-1*(*qm150*) *IV*; *ctb-1*(*189*) *M* double mutant, also obtained from the CGC and integrated onto our N2 wildtype nuclear strain background. Following standard *C. elegans* nomenclature, *IV* refers to the nuclear chromosome number and *M* refers to the mitochondrial genome in the double mutant name.

**TABLE 1:** Strains, affected ETC proteins, and mating system. Apart from the *gas-1* and the *isp-1 V; ctb-1* *M* double mutants, we utilized strains containing each of four ETC mutations (two mtDNA-encoded, and two nDNA encoded) on three genetic backgrounds affecting mating system: wildtype (facultatively outcrossing), *xol-1* (obligately selfing), and *fog-2* (obligately outcrossing). Because results from a facultatively outcrossing *gas-1* mutant (gray highlight) was previously reported in Wernick et al. (2019), it was not included in the current study. For logistical reasons, we also only studied a facultatively outcrossing version of the mitochondrial-nuclear double mutant, *isp-1 V; ctb-1 M*. Following standard *C. elegans* genetic nomenclature for such double mutants, the nuclear gene name is shown first alongside the chromosome number, followed by the mitochondrial gene name and *“M”* to denote the mitochondrial chromosome.

Strain	Mitochondrial ETC Location	Mating System
N2	wildtype	Facultatively outcrossing
*cox-1*	ETC complex IV (mtDNA)	Facultatively outcrossing
*xol-1 X*; *cox-1 M*	ETC complex IV (mtDNA)	Obligately selfing
*fog-2 V*; *cox-1 M*	ETC complex IV (mtDNA)	Obligately outcrossing
*ctb-1*	ETC complex III (mtDNA)	Facultatively outcrossing
*xol-1 X*; *ctb-1 M*	ETC complex III (mtDNA)	Obligately selfing
*fog-2 V*; *ctb-1 M*	ETC complex III (mtDNA)	Obligately outcrossing
*isp-1*	ETC complex III (nDNA)	Facultatively outcrossing
*xol-1*; *isp-1*	ETC complex III (nDNA)	Obligately selfing
*fog-2*; *isp-1*	ETC complex III (nDNA)	Obligately outcrossing
*gas-1*	*ETC complex I (nDNA)*	*Facultatively outcrossing*
*xol-1*; *gas-1*	ETC complex I (nDNA)	Obligately selfing
*fog-2*; *gas-1*	ETC complex I (nDNA)	Obligately outcrossing
*isp-1 IV*; *ctb-1 M*	ETC complex III (nDNA) and complex III (mtDNA)	Facultatively outcrossing

The nuclear-encoded *gas-1* gene, so named for its general anesthetic hyper-sensitive response, is located on the X chromosome and encodes GAS-1, a core 51 kDa protein subunit (orthologue of NDUFS2 in human and mouse and the bovine 49 kDa subunit) of mitochondrial ETC complex I that is required for oxidative phosphorylation (Kayser et al., 1999). Mutations of this gene are associated with severe phenotypic consequences including reduced progeny production, reduced complex I-dependent metabolism, hypersensitivity to oxidative stress owing to increased ROS production, and low ATP levels relative to wildtype (reviewed in Van Der Bliek et al., 2017).

The nuclear *isp-1* gene encodes the Rieske iron-sulfur protein of ETC complex III (cytochrome bc1 complex), which transfers electrons from ubiquinol to cytochrome c and, like complex I, simultaneously pumps protons across the mitochondrial inner membrane thereby helping to establish the proton gradient. Other *isp-1* mutants have been characterized by slowed metabolism and extended lifespan (Rea, 2005; Ventura et al., 2006). The slow-living phenotype of *isp-1* appears to result from an increased reliance upon alternate, and less efficient, metabolic pathways for energy production and a concomitant reduction in mitochondrial respiration (Rea, 2005; Jafari et al., 2015).

In addition to an *isp-1* deletion generated *via* CRISPR-Cas9, we utilized a previously characterized *isp-1 IV; ctb-1 M* double SNP mutant (Feng et al., 2001). The mutant screen of Feng et al. (2001) identified *isp-1(qm150)* and also yielded an *isp-1(qm150) IV*; *ctb-1(189) M* double mutant. We first isolated the nuclear *isp-1* mutant allele onto our laboratory wildtype N2 strain *via* 10 rounds of backcrossing; males were then mated to hermaphrodites of the mitochondrial *ctb-1* mutant strain prior to use in the current experiments. ISP-1 and the mtDNA-encoded cytochrome b, CTB-1, physically interact within ETC complex III. This *isp-1* mutation replaces a conserved proline with a serine in the head domain of ISP-1, which functions to transfer reducing equivalents within complex III to cytochrome c1 through a series of conformational changes (Iwata et al., 1998). Homology modeling indicates that this mutation distorts the structure and alters the redox potential of ISP-1 (Feng et al., 2001; Jafari et al., 2015). *ctb-1(qm189)* is a homoplasmic (fixed within and across individuals in a population) allele that substitutes a valine for a conserved alanine in CTB-1 near the binding site of the ISP head domain (Feng et al., 2001). The *isp-1* and *ctb-1* mutant locations are not predicted to directly interact (Iwata et al., 1998). However, *ctb-1(qm189)* partially suppresses the *isp-1(qm150)* phenotype *via* beneficial allosteric effects on complex I (Suthammarak et al*.,* 2009) This finding makes sense in light of the fact that ETC complexes I, III and IV form stable supercomplexes that improve ETC functionality (Acín-Pérez et al., 2008). *isp-1(qm150)* weakens the association of this supercomplex and reduces the amount and activity of complex I. The *ctb-1(qm189)* mutation exhibits sign epistasis (Weinreich et al., 2005) as it is beneficial within the context of *isp-1(qm150)*, but by itself causes slightly deleterious effects on fitness and complex III activity (Suthammarak et al., 2009).

The *cox-1* gene encodes cytochrome c oxidase I (COX-1), the main catalytic subunit of cytochrome c oxidase or ETC complex IV. The three mtDNA-encoded subunits (COX-I, II, III) form the functional core of the complex; the nDNA-encoded subunits are essential for complex assembly and function (Barrientos et al*.,* 2002; Li et al., 2006). Many additional nDNA genes are essential for biogenesis of the functional complex (Barrientos et al*.*, 2002), and reduce oxygen to water. It is not believed to be a major contributor to ROS production. However, its structural/functional state may indirectly affect ROS generation *via* other members of the I:III:IV supercomplex (Greggio et al., 2017). Cytochrome c oxidase deficiency is a leading cause of human mitochondrial disorders (Shoubridge, 2001), and interpopulation (Rawson and Burton, 2002) and interspecies (Sackton et al., 2003) hybrid incompatibilities are documented to result from breakup of coadapted gene complexes involving this enzyme. We used a *cox-1* mutation isolated from a wild *C. elegans* strain, CB4856 (Hawaii) (Dingley et al., 2014). The mtDNA of this strain differs from that of the laboratory wildtype N2 by a substitution that replaces an alanine with a serine in the N-terminus of COX-1 within the matrix side of the complex IV catalytic core. Interestingly, the variant was found to be beneficial (i.e., cause increased mitochondrial membrane potential) to CB4856 worms cultured at their native temperature of 25°C, but exerted a variety of deleterious effects at the standard laboratory temperature of 20°C (i.e., reduced lifespan, elevated mitochondrial matrix oxidant burden, and oxidative stress). A transmitochondrial cybrid strain containing a CB4856 mtDNA genome (homoplasmic for the *cox-1* variant) on a N2 nDNA background exhibited similar deleterious phenotypes at 20°C (Dingley et al., 2014). Later studies including that by Zhu et al. (2019) found that CB4856 mitochondria were also associated with reduced fecundity in the presence of an N2 nuclear background.

*Culture conditions.* Eight replicate populations of each ETC ancestral mutant strain (henceforth referred to as G0 for generation 0) on each of the three genetic backgrounds underwent laboratory evolution following our previous methods (Wernick et al., 2019) wherein replicate “recovery lines” (RC lines; c. f., Estes and Lynch, 2003) were maintained under standard laboratory conditions but in large population sizes (bottleneck sizes of 1,000) for at least 60 generations, resulting in sets of “G60 RC lines”. Eight replicates of the N2 control strain underwent the same treatment. Populations were maintained at 20°C on 100 mm Petri plates containing Nematode Growth Medium Light (NGML), 1 ml of 200 mg/ml streptomycin, and streptomycin-resistant OP50-1 *Escherichia coli* as a food source. For each RC line, standardized bleach treatment was used to maintain evolving populations in non-overlapping generations. Each generation, worms were rinsed from crowded plates using M9 buffer into 15 ml conical tubes. Conical tubes were centrifuged at 800 rpm for 30 s and excess M9 poured off. A mixture of three parts diluted commercial bleach (final concentration = 2.75% bleach in di H_2_O) and one part 5M NaOH was then added to the conical tubes. Tubes were inverted every 2 mins until the worms were dissolved, releasing viable embryos. Once dissolved, the conical tubes were centrifuged again until an embryo pellet formed. The bleach, NaOH and M9 were poured off and fresh M9 added to rinse the embryo pellet. This rinse process was repeated three times. The embryo pellet was then transferred to a 1 ml microtube and vortexed, after which 1 ul was transferred onto an eight-well slide. Embryo counts were used to calculate the amount needed to transfer 1,000 individuals onto new large plates to initiate the next generation. Strains were transferred when the majority of hermaphrodites or females reached peak gravidity and began laying embryos with a few hatched larvae; plates were typically well-starved by this point. The time in days between transfers was tracked for all lines across the experiment.

*Fitness assays.* Following standard protocols, we assayed fitness for all *cox-1*-containing lines alongside the wildtype N2 control in order to more fully interpret any observed changes in male frequency and mating-related traits. For all facultatively outcrossing (N2 background) and obligately selfing (*xol-1* background) lines, we assayed daily production of selfed progeny following established methods (e.g., Wernick et al., 2019). These assays were initiated by allowing 10–15 adult hermaphrodites from an individual line to lay a pool of embryos for 5 h. Single embryos were then transferred to individual 60 mm Petri plates containing NGML, 1 ml of streptomycin, and OP50-1 *E. coli* food source, and allowed to develop. Once hatched, the number of plates was reduced to 20 for the N2 control, 10 for each ancestral mutant, and five for all G60 RC lines. At the same time each day, hermaphrodite parents were transferred to a fresh plate. Offspring were allowed to develop to the L3/L4 larval stage and then killed with a drop of 0.5 M sodium azide and stored at −4°C to be counted. Offspring were counted by counterstaining plates with toluidine blue dye.

Outcrossed progeny production was assayed for both the facultatively outcrossing *cox-1* lines (N2 background) and the obligately outcrossing *fog-2 V; cox-1 M* lines. These assays were initiated by picking individual L4 larval stage male and female pairs onto fresh plates, 20 pairs for the ancestral mutant and 10 pairs for all G60 RC lines. The focal pairs were transferred together every 24 h and offspring were counted as described above.

Offspring counts from both selfed- and outcrossed-fitness assays were used to generate reproductive schedules and calculate total reproductive output and relative fitness of the *cox-1* G0 mutant compared with N2, and with each of the *cox-1* G60 RC lines following (Christy et al., 2019). Only progeny counts from pairings that produced male offspring (which signaled a successful mating) were included in analyses for outcrossed progeny production. Relative fitness of each individual was computed as: *ω* = *Σe* ^*-rx*^ *l(x)m(x)*, where *l(x)* is the number of worms surviving to day *x*, *m(x)* is the fecundity at day *x*, and *r* is the mean intrinsic population growth rate of the assay-specific N2 or *cox-1* G0 control as appropriate. The latter was calculated by solving Euler’s equation for *r* from *ω* = *Σe* ^*-rx*^ *l(x) m(x)* = 1 using an average value of *l(x) m(x)* for each block-specific control. We used *x* = 4.75 on the first reproductive day (cf., Vassilieva et al., 2000).

*Male frequency.* Male frequency counts were conducted for each RC line at G5, G10 and every ten generations up to 60 following the methods of Katju et al. (2008). Such counts were also conducted for obligately outcrossing (*fog-2*) and obligately selfing (*xol-1*) strains (with expected male frequencies of 50% and 0%, respectively), for control comparison and to regularly ensure against cross-contamination of evolving lines. Two samples of approximately 200 individuals from each RC line were plated onto two 60 mm Petri plates. In a small number of instances, only one sample could be counted. The number of males and hermaphrodites (or females for *fog-2* lineages) on each plate were counted to calculate an average ratio of males for each RC line.

*Mating behavior.* To evaluate biological changes accompanying male frequency evolution, we measured several phenotypes including male and hermaphrodite/female mating behaviors. Mating behavior assays were performed for both *cox-1* and *fog-2 V; cox-1 M* G0 strains and their G60 RC lines. Replicate G60 lines were thawed along with their G0 progenitor strains and allowed to recover for three to four generations. Unmated L4 hermaphrodites (*cox-1*) or females (*fog-2 V; cox-1 M*) were isolated approximately 24 h prior to assays. For the assays, single unmated hermaphrodites or females were picked to individual 35 mm plates with small (approximately 1 cm diameter) OP50-1 *E. coli* lawns, and four young males were picked to the plate near them. The plates were then observed every 5 min for 60 min and each male was assigned a score according to the following ethogram developed based on the highly stereotyped mating behavior of *C. elegans* males (Barr and Garcia, 2006): *1* = at least one instance of male contact with hermaphrodite/female, male nose search; *2* = backward male locomotion, tail search; *3* = male turning around hermaphrodite/female body; *4* = at least one instance of vulval location; male tail prodding; *5* = at least one instance of male spicule insertion. The number of males actively courting the hermaphrodite/female were also recorded for each 5 min interval. Lastly, hermaphrodite/female latency to mate, where mating was defined as the first observation of male spicule insertion and attachment for at least 4–5 s, was also recorded to the nearest second. Assays were conducted in batches of eight replicates per 1 h period, and included four G0 replicates and four G60 replicates per RC line.

The distribution of hermaphrodite/female latency to mate data was quite platykurtic with a peak at 60 min, the time at which observations ended; no transformation achieved normality. Consequently, we performed a Van der Waerden test followed by non-parametric Steel-Dwass comparisons of the *cox-1* and *fog-2 V; cox-1 M* G0 and RC line groups.

*Male mating success.* We measured male mating success in ancestral G0 mutant and RC lines following methods similar to those of Teotónio et al. (2006) except that we evaluated the ability of males to mate successfully with both tester *fog-2* females obtained from the CGC and to coevolved hermaphrodites (*cox-1*) or females (*fog-2 V; cox-1 M*) from their own strain and generation*.* The *xol-1* strains were necessarily omitted from these analyses. Virgin G60 RC males and ancestral G0 mutant males were picked from age-synchronized populations individually onto 35 mm Petri plates containing NGML, 1 ml of 200 mg/ml streptomycin, and OP50-1 *E. coli*. We then paired single experimental males with single virgin L4 *fog-2* tester females, and separately with either single virgin L4 hermaphrodites or single females from the male’s same strain and generation (i.e., G0 or G60). Pairs were given 24 h to mate; the experimental male was then removed from the plate. After an additional 24 h, the plate was examined for the presence of embryos and the mating was initially recorded as a success or failure on this basis. To confirm whether matings were successful for the *cox-1* lines, each hermaphrodite was transferred onto a fresh plate for two or more additional days. Offspring were allowed to hatch and mature until the L4 stage, then killed with a drop of 1 M sodium azide and inspected for males. If male offspring were observed, the mating was considered successful; if no male offspring were present, the mating was designated as unsuccessful. Mating success was analyzed using a generalized linear model with 3-way factorial design to test the effects of mating system, treatment (G0 versus G60 RC), and mate source (coevolved hermaphrodite/female versus *fog-2* tester female), with line included as a random variable (glmm package in R v. 3.6. 2).

*Outcrossing ability*. We also measured the rate of outcrossing for the pairings involving experimental males and coevolved hermaphrodites or females (in the case of *fog-2* strains) from their same ancestral G0 or RC lineage as described above*.* These assays, from which the *xol-1* strains were necessarily omitted, were performed independently of the fitness assays. Experimental pairs involving either G0 males and hermaphrodites or females, and G60 males and hermaphrodites or females were given 24 h to mate; the experimental male was then removed from the plate. Each hermaphrodite or female was transferred onto a fresh plate every 24 h for 2 days. Offspring were allowed to hatch as above and male and hermaphrodite/female frequency was recorded. A ratio of males was created by dividing the number of males by the total offspring population size. To quantify outcrossing rates, we applied the formula 2 (*m-μ*) (Morran et al., 2009b), where *m* is equal to offspring male frequency and *μ* is equal to rate of X chromosome nondisjunction, which was evaluated as previously described (Wernick et al., 2019). Following previous *C. elegans* studies, our measure of average outcrossing rate accounts for failed matings, in which outcrossing rates would necessarily equal zero.

*Male sperm traits*. We examined traits related to *C. elegans* sperm competitiveness—sperm area and number—in G60 *cox-1* RC males that experienced evolution of male frequency, alongside the obligately sexual *fog-2* RC lines and the G0 mutant and wildtype N2 controls. The *cox-1* line RC3 was omitted from analysis owing to the impossibility of maintaining males as described later. For all other lines, virgin males were separated from hermaphrodites or females, and picked into a microtubule containing 495 ul of M9 buffer. When approximately 40 males were transferred, 5 ul of 1 mM MitoSox Red (Thermo Fisher Scientific, Waltham MA, United States) diluted in DMSO was added to the tube, which was then placed in the dark for 2 h. Once staining was complete, males were transferred using a glass pipette onto a 60 mm Petri plate and allowed to recover overnight. The following day, the stained sexually mature virgin males were plated individually onto a 35 mm Petri plate with one anesthetized young adult virgin *fog-2* tester female that had been separated from a mixed population at the L4 stage the day prior. The tester females were anesthetized by first being picked into a microtubule of 71.2 ul of M9 buffer; 8 ul of a 1% tricane stock solution and 0.8 ul of a 1% of a tetramisole hydrochloride stock solution of was then added to the microtubule. After 45 min, tester females were removed using a glass pipette and placed onto mating plates where they were allowed to mate with virgin experimental males for 80 min in the dark. After mating, the tester females were mounted on an agar slide, which were created placing a dime-size amount of agar mixture (1 g of agar and 10 ml of M9 buffer, microwaved for 15 s) onto a glass slide. After mating with stained males, the tester females were picked onto these slides and a glass cover slide was placed on top. Labeled male sperm inside the translucent, mated tester females were then visualized using a Leica DM2500 confocal fluorescence microscope. To capture all sperm transferred, linear Z-compression images of 0.35 um were taken for each worm, and the images joined together in a Z-stack allowing for visualization through the entire worm.

Confocal micrographs were viewed using ImageJ software v. 1.52a (National Institutes of Health). The number of male sperm transferred during copulation was also recorded utilizing a tally counter application to count all fluorescing sperm throughout the Z-stack. Measurements of individual cells involved importing batches of images with a scale bar of 25 um. The scale and global settings were set to um with a known distance of 25 um, pixel aspect ratio of 1.0. The widest portions of 10 randomly-selected cells per animal were captured in pixels/um (length), followed by a second measurement in the opposite dimension (width). Due to logistical constraints, we measured cross-sectional area on the same cells, calculated using the equation π × (length/2) × (width/2), and an average of the 10 calculated sperm areas was recorded for each animal. We note that this method differs from, and is likely inferior to, those of other studies which typically measure the size of spermatids released from dissected male gonads into sperm medium on Petri plates (e.g., Ting et al., 2018). These immature cells will conform more closely to the elliptical shape assumed by the equation.

*Data analysis*. Unless otherwise noted, we first compared traits among the various G0 ancestral strains: wildtype N2, *fog-2*, *xol-1*, *cox-1* (assayed by both selfing and outcrossing), *fog-2 V; cox-1 M*, and *xol-1 X; cox-1 M* using a one-way ANOVA followed by Tukey’s HSD comparisons among all pairs of strains. We then evaluated the model: *trait* = *μ* + mating system + treatment + (mating system × treatment) + line + *ε*, where mating system (N2, *fog-2*, or *xol-1*, corresponding to wildtype facultatively outcrossing, outcrossing, and selfing) and treatment (G0 ancestor versus G60 RC) are fixed effects and line is a random effect, using restricted maximum likelihood (REML) with the mixed procedure of JMP Pro (v. 13.0, SAS Institute). To test whether traits of each RC line differed from the levels in the appropriate G0 ancestor, we constructed contrasts using the model trait = line. We evaluated the difference in fitness-related traits between the G60 *cox-1* RC lines assayed by selfing and outcrossing using the model: *trait* = *μ* + mating system + line + (mating system × line) + *ε*. We compared the N2 G0 and its facultatively outcrossing RC lines assayed by self-crossing to those assayed by outcrossing in a separate analysis.

## Results

**FIGURE 1:**
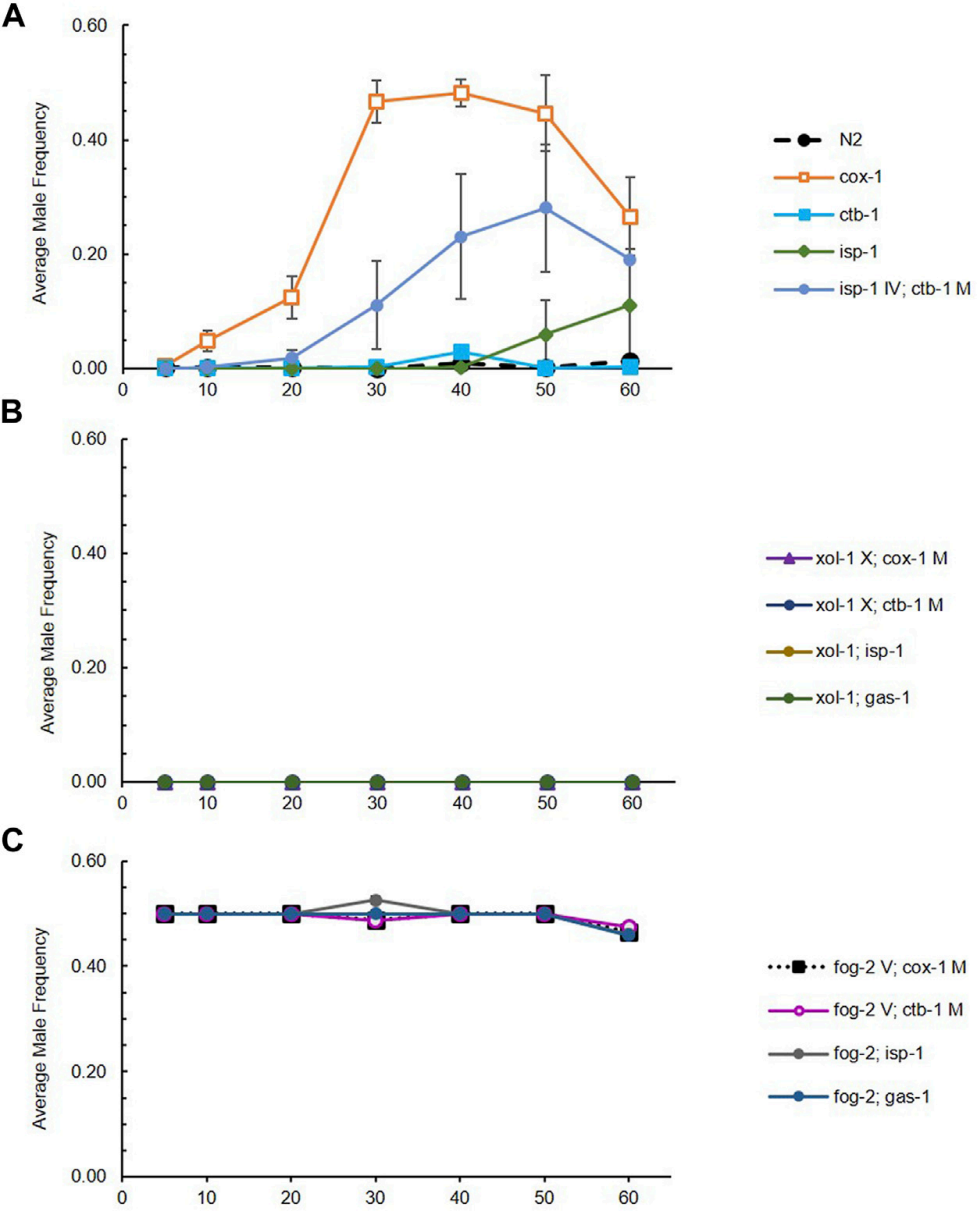
Average male frequency of RC line sets (8 lines each) generated from ETC mutant/mating system combinations across 60 generations. **(A)** Average male frequency of RC line sets generated from wildtype N2 and ETC mutants on otherwise wildtype (facultatively outcrossing) genetic backgrounds. The orange line represents evolution of male frequency in the *cox-1* RC line set. Note that male frequency of *gas-1* lines were reported in Wernick et al. (2019) and that this strain was not re-evolved here. **(B)** Average male frequency of RC line sets generated from ETC mutants on *xol-1* (obligately selfing) genetic backgrounds. **(C)** Average male frequency of RC line sets generated from ETC mutants on *fog-2* (obligately outcrossing) genetic backgrounds. Bars represent 95% confidence intervals. Some lines at 0 and 50% are not visible.

**FIGURE 2:**
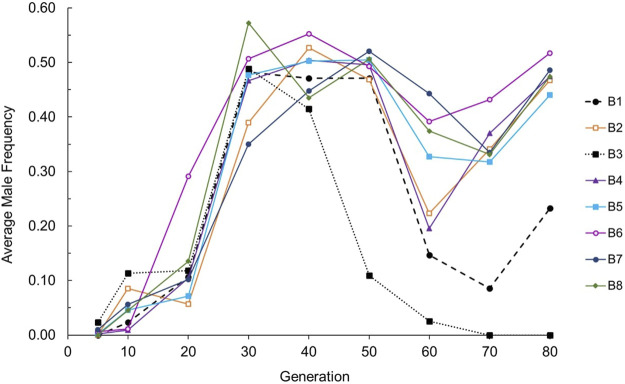
Average male frequency across 80 generations for the eight independently-evolved *cox-1* RC lines. The dashed line represents RC line B1; the dotted line represents RC line B3.

*Evolution of male frequency.* We first evaluated the change in male frequency across generations in each strain (wildtype N2 or ETC mutant), each evolving within the context of three mating systems. Male frequency was stable across experimental generations for obligately outcrossing (approximately 50%) and obligately selfing (0%) strains as expected, but exhibited statistically significant evolutionary change for at least one generational timepoint in at least one replicate RC line from all facultatively outcrossing strains: *cox-1, ctb-1, isp-1*, and *isp-1 IV; ctb-1 M* (two-tailed *t*-tests comparing male frequency at a given generation to that at G5 for each RC line; see Supplementary Table S1 for raw data, and Figure 1 for averages across replicate lines for each strain). There was no tendency for mitochondrial or nuclear ETC mutants to be associated with higher maximum male frequencies (Figure 1A). All *cox-1* RC lines exhibited a fairly uniform increase in male frequency until approximately G40, after which point average male frequency began to decline and then later increased again at G80 (Figure 2). Later generations also saw increased variance among *cox-1* RC lines, with lines B1 and B3 showing substantial declines in male frequency between G40-50 and G70, and RC line B3 completely losing males by G70. By contrast, only one *ctb-1* RC line, E8, exhibited a short-lived increase in male frequency, transitioning from no males to 18% males at G40 (a statistically significant increase from zero males at G5; *t*_*1*_ = 21.35, *p* = 0.002), then falling to 6% and 0% at G50 and G60, respectively. Two of the eight *isp-1* RC lines, H4 and H8, exhibited elevated male frequency starting at generation 50. For H4, average male frequency was 11% at G50 and 52% at G60. For H8, average male frequency was 36% at both G50 and G60. Finally, patterns of male frequency evolution in the *isp-1 IV; ctb-1 M* double mutant were somewhat similar to those of *cox-1*; here six replicate lines—M1, M2, M5, M6, M7, and M8—evolved higher-than-wildtype levels of male frequency (Supplementary Figure S1). One line, M2, achieved a smaller but still statistically significant maximum male frequency of 12% at G30 (*t*_*1*_ = 10.82, *p* = 0.008), followed by a slow decline to 0% at G60. The other *isp-1 IV; ctb-1 M* RC lines exhibited increased frequencies, which peaked at 30–53% at G50 and then declined at G60.

Imaging of a single, mature *cox-1* B3 line male recovered from the G60 population revealed phenotypically normal gross morphology and a large number of spermatocytes within the gonad. However, we noticed during this and other attempts to recover and maintain B3 line males that they appeared to develop much more slowly than hermaphrodites from the same line. Although we did not closely track developmental timing, we observed that B3 males were still very small, approximately the same size as a typical wildtype L2-stage animal, when B3 hermaphrodites from the same brood had reached the young adult stage (i.e., reproductive maturity). The B3 males exhibited no ability for successful mating until approximately 1.5 days after hermaphrodites had reached reproductive maturation. Such sex-specific variation in developmental timing within a lineage would be expected to cause a rapid loss of males from a population.

*Fitness.* To shed light on how *cox-1*-induced mitonuclear mismatch and mating system may have influenced the evolution of relative fitness and associated life-history traits, we compared phenotypes of G0 and G60 animals from each strain. There was no need to account for a block effect in analyses of fitness data as mean total offspring production by the N2 control strain did not vary significantly across assay dates. Similarly, as a group, RC lines initiated from N2 exhibited no evolution across 60 generations for *ω* or progeny production (“A” RC lines in Supplementary Table S2).

**FIGURE 3:**
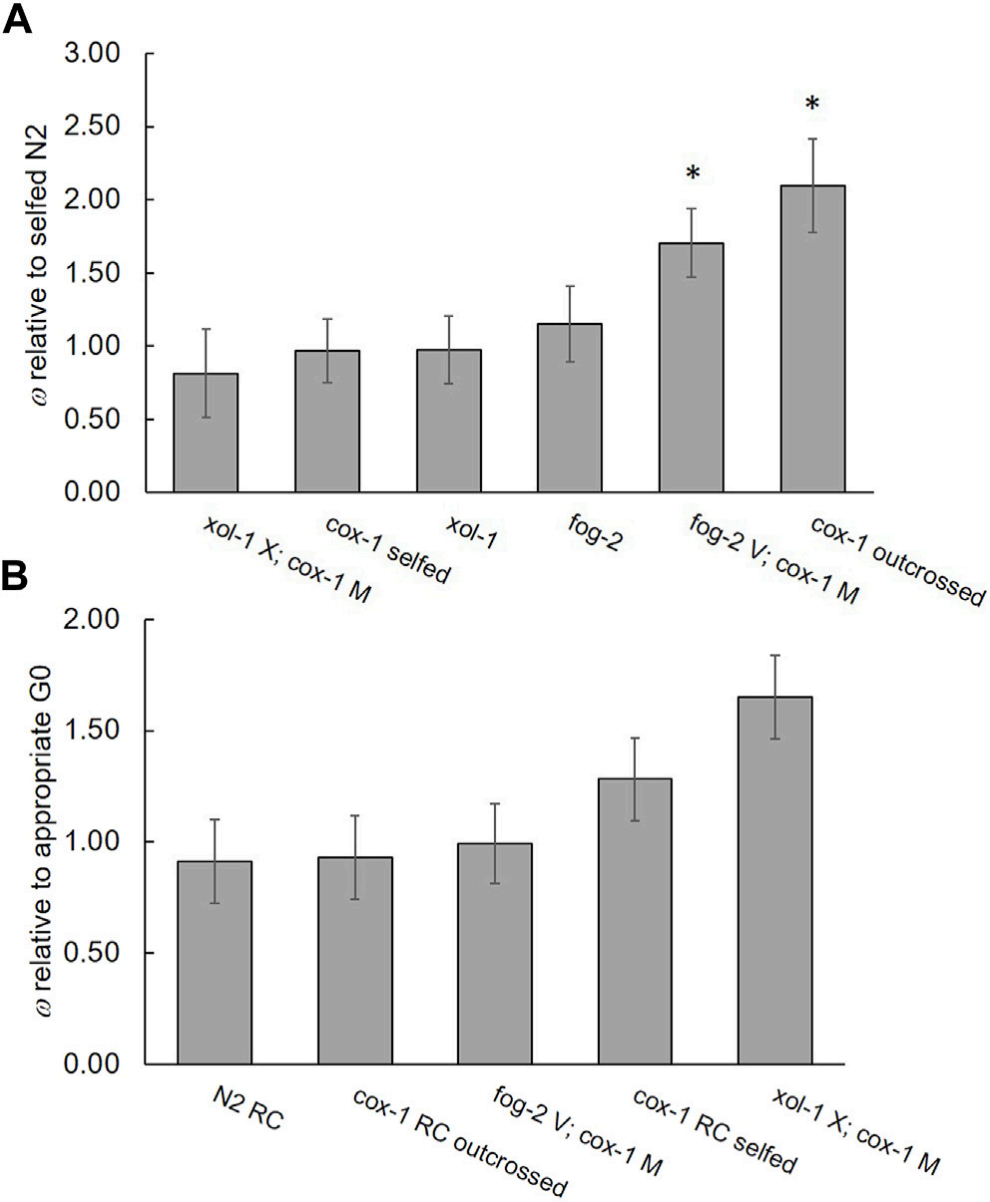
Relative fitnesses. **(A)** Fitness of G0 ancestral mutant strains relative to self-crossed wildtype N2; arranged by increasing mean relative fitness. Asterisks denote statistically significant differences between G0 ancestral mutants and N2. **(B)** Fitness of RC line groups relative to their appropriate G0 ancestor. *n* = 8 lines per group except *cox-1* RC outcrossed where line B3 was removed owing to an absence of males. Note the change in *y*-axis scales. Bars represent 95% confidence intervals.

We detected large, statistically significant differences in *ω* measured relative to N2 among the ancestral strains of *fog-2*, *xol-1*, *cox-1* (assayed by both selfing and outcrossing), *fog-2 V; cox-1 M*, and *xol-1 X; cox-1 M* (*F*_*6*_ = 15.64, *p* < 0.001), with *fog-2 V*; *cox-1 M* and outcrossed *cox-1* exhibiting higher fitness than the other G0 strains (Tukey’s HSD, *α* = 0.05) (Figure 3A; Supplementary Table S2). The increased relative fitness of these two strains was due particularly to their increased early-life productivity compared to other strains (Supplementary Table S2). Mean early-life productivity ± 1 S.E.M. = 110.3 ± 15.35 for *fog-2 V; cox-1 M* and 152.5 ± 13.10 for outcrossed *cox-1*, versus only 72.20 ± 2.800 for wildtype N2. Regarding the evolution of fitness in the RC lines, only the treatment was marginally significant for *ω* measured relative to the appropriate G0 ancestor (*F*_*1*_ = 4.26, *p* = 0.057). Specifically, among the *cox-1* lines assayed by selfing, two RC lines (B6 and B7) were significantly more fit than the G0, and B3 was marginally less fit than G0 (*p* = 0.08) (Dunnett’s test; *d* = 2.81, *α* = 0.05 Supplementary Table S2). Among the *fog-2 V; cox-1 M* lines, no individual RC line exhibited a change in fitness relative to the G0 (Dunnett’s test; *d* = 2.77, *α* = 0.05). Among the *xol-1 X; cox-1 M* lines, all RC lines except D3 had improved fitness compared to the G0 (Dunnett’s test; *d* = 2.83, *α* = 0.05). Figure 3B shows the average *ω* for each RC line set.

A comparison of the *cox-1* lines evaluated by selfing versus outcrossing revealed the expected pattern of increased productivity with outcrossing. RC line B3 was removed from this analysis since males could not be maintained to perform the outcrossing assay. Fitness relative to the appropriate (selfed or outcrossed) *cox-1* G0 strain, was affected by mating system (*F*_1_ = 11.49, *p* < 0.001), treatment (G0 or G60 RC) (*F*_*1*_ = 5.992, *p* = 0.016) and their interaction (*F*_1_ = 11.50, *p* < 0.001). Specifically, the *cox-1* outcrossed lines produced more offspring (365) compared to the *cox-1* selfed lines (243) as expected given that *C. elegans* hermaphrodites are sperm limited. However, while the *cox-1* outcrossed RC lines showed no significant change in average *ω* (± 1 S.E.M.) compared to the outcrossed *cox-1* G0 (*ω* = 0.93 ± 0.038), the same lines assayed by selfing showed upward evolution of *ω* (1.40 ± 0.051) compared to the selfed *cox-1* G0. In other words, *cox-1* hermaphrodites’ selfed but not outcrossed fitness increased during laboratory evolution. Among the *cox-1* outcrossed RC lines, only RC line B5 exhibited different (lower) fitness compared to the G0 (Dunnett’s test; *d* = 2.69, *α* = 0.05).

**FIGURE 4:**
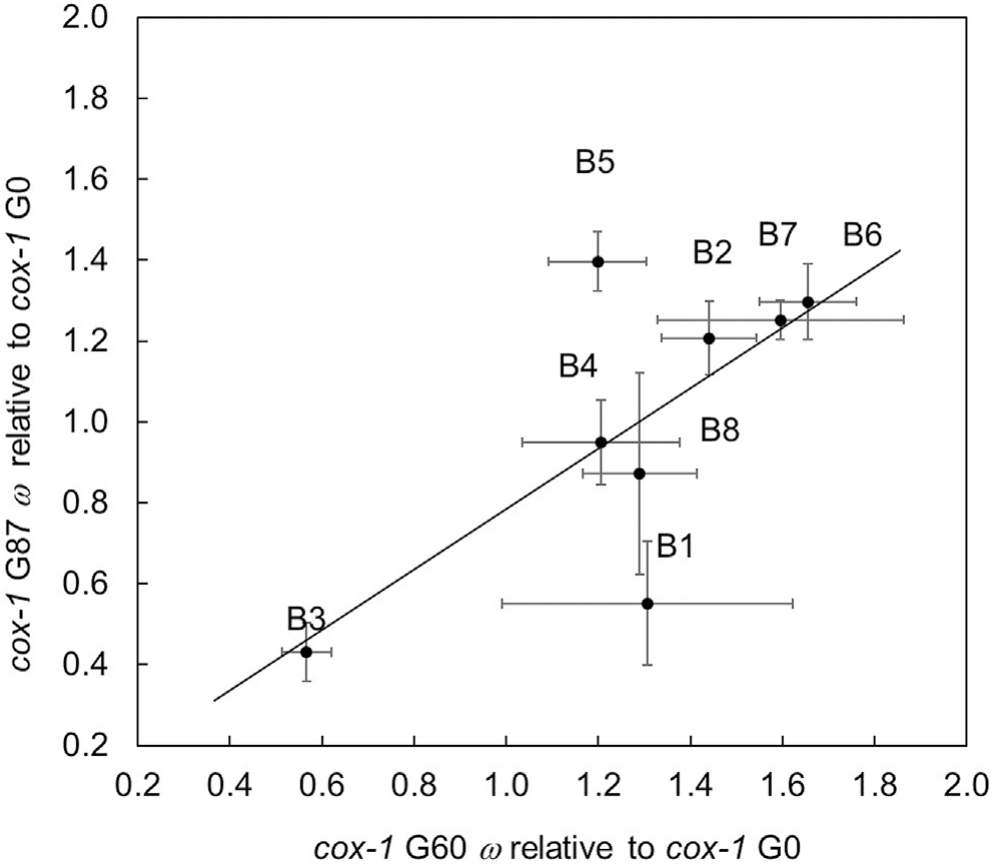
Fitness of self-fertilized *cox-1* G60 and G87 RC lines relative to their G0 ancestral mutant strain. Error bars = 1 S.E.M. The best-fit line of the data using standard least squares regression where *y* is G87 fitness and *x* is G60 fitness: *y* = 0.7472(*x*) + 0.0362 (*F*_1, 8_ = 5.78, *p* = 0.053, nonsignificant intercept); *R* ^2^ = 0.490.

Owing to obvious changes in male frequency among the *cox-1* RC lines, we elected to extend the evolution experiment for these lines to 87 total generations and compare self-crossed fitness at both time points. A test of the model *ω* = *μ* + generation + line + (generation × line) + *ε* revealed a significant effect of generation (*F*_*1*_ = 9.52, *p* = 0.019) such that average *ω* (± 1 S.E.M.) of G60 *cox-1* RC lines (1.28 ± 0.123) was higher than that of the same lines at G87 (0.995 ± 0.124). Figure 4 compares the selfed fitness of each *cox-1* RC line relative to its *cox-1* ancestor at G60 and G87. With only one exception, RC line B5, the selfed fitness of G87 RC lines showed a tendency to be lower than at G60. Average relative fitnesses of lines at each generation were marginally significantly positively correlated (*r* ^2^ = 0.700, *p* = 0.053), but the upper 95% confidence interval for the estimate did not overlap 1.0 (95% *CIs* = −0.008, 0.941).

*Mating behavior.* We also evaluated the effect of mating system on both hermaphrodite/female mating avoidance and the intensity of male mating behavior in the *cox-1* and *fog-2 V; cox-1 M* mutant lines. Hermaphrodite/female latency to mate varied significantly across *cox-1* and *fog-2 V; cox-1 M* G0 and RC groups (Van der Waerden test; *χ* ^2^_3_ = 20.15, *p* < 0.001) such that *cox-1* lines had higher average latency to mate than *fog-2 V; cox-1 M* lines (46.7 min compared to 26.8 min, respectively), (Steel-Dwass comparisons; *q* = 2.569, *α* = 0.05). However, neither set of RC lines differed significantly from their G0 ancestor. Male courtship data were noisy, but revealed that *fog-2* lines were slightly more vigorous courters based on the fraction of males courting at an ethogram score of two or above across the assay, and particularly during the earliest time points. For instance, at 10 min timepoint, the *cox-1* and *fog-2 V; cox-1 M* line groups differ significantly in the number of males (out of 4) courting at this level or above (*t* = −3.274, *p* < 0.001). Specifically, at this time point, an average (± 1 S.E.M.) of only 0.41 ± 0.21 *cox-1* males were courting at this intensity level compared to 1.3 ± 0.17 *fog-2 V; cox-1 M* males. There was no obvious tendency for either set of RC lines to differ from their G0 ancestors in male courtship intensity.

*Male mating success.* Mating trials were performed to determine the ability of G0 and G60 experimental *cox-1* and *fog-2 V; cox-1 M* males to successfully mate with both coevolved and tester females. Whether males were successful at mating (Y/N) in our assay was significantly influenced only by the interaction of sexual system (N2 or *fog-2*) and mate source (coevolved versus tester) (*p* < 0.05), but not by either of these factor or treatment (G0 or G60) as a main effect, in a test of the full model. Specifically, *cox-1* G0 males had a very low level of mating success with *fog-2* tester females (13% on average) at G0, and evolved greater success over the experiment (31% on average in G60 RC lines). Conversely, *fog-2 V; cox-1 M* G0 males had relatively high level of initial success with *fog-2* testers at G0 (68% on average) but performed worse by G60 (34% in G60 RC lines) (Supplementary Figure S2). In contrast, both *cox-1* and *fog-2 V; cox-1 M* males had high levels of mating success with their coevolved hermaphrodites/females (75% and 79%, respectively), at G0, and both evolved lower mating success with these coevolved mates by G60 (55% and 53%, respectively, across their G60 RC lines).

*Outcrossing ability.* We evaluated the influence of *cox-1*-induced mitonuclear mismatch and mating system on the ability of coevolved male and hermaphrodite/female pairs to produce outcrossed offspring. Mean outcrossing rates for pairings from N2, *cox-1,* and *fog-2 V*; *cox-1 M* line sets are displayed in Supplementary Table S2. There was no significant difference in the outcrossing rates between the N2 control and the *cox-1* G0 ancestor (mean ± 1 S.E.M. = 0.369 ± 0.037 versus 0.250 ± 0.103, respectively). As expected, the obligately outcrossing *fog-2 V; cox-1 M* ancestor exhibited high estimated rates of outcrossing (0.768 ± 0.103) relative to the N2 control. These measurements were unaffected by elevated rates of X-chromosome nondisjunction, which were essentially zero for all strains (Supplementary Table S2). In line with previous findings, outcrossing rates were variable among individuals within lines and exhibited unequal variance across groups. A Welch’s ANOVA found significant variation in outcrossing among the *cox-1*, *fog-2 V; cox-1 M* G0 and RC line groups (*F*_3_ = 8.103, *p* = 0.0002) such that *fog-2 V; cox-1 M* lines had higher outcrossing rates than *cox-1* lines—as expected given that the *fog-2* strain is obligately outcrossing. As a reminder, the measure of outcrossing rate takes failed matings into account, so would not necessarily be expected to equal 100% for *fog-2*-containing lines. Within the *cox-1* lines, there was no significant change in outcrossing rates in the G60 RC lines (0.364 ± 0.035) compared to the G0 ancestor (0.398 ± 0.089). However, *cox-1* RC line B3 was again removed from this analysis owing to the difficulty of maintaining males; the outcrossing rate for this line is likely to be zero. Therefore, the true value for *cox-1* G60 RC line outcrossing is lower than we are able to estimate. Within the *fog-2 V; cox-1 M* RC lines, the G60 RC lines as a group exhibited reduced outcrossing compared to their G0 ancestor (0.506 compared to 0.768; Welch’s *t* = 6.771, *p* = 0.016).

*Male sperm traits.* Finally, we measured the evolution of male sperm size and the number of sperm transferred during copulation for both *cox-1* and *fog-2 V; cox-1 M* males. We found significant differences among the ancestral G0 strains of N2, *cox-1*, and *fog-2 V; cox-1 M* in both average male sperm size (*F*_2_ = 9.206, *p* < 0.001) and the number of sperm transferred (*F*_2_ = 13.285, *p* < 0.0001) (Supplementary Table S2). In particular, *fog-2 V; cox-1 M* G0 males had larger sperm (8.461 um^2^ on average) than males of either N2 (5.442 um^2^) or *cox-1* (5.204 um^2^) ancestral strains, and both *cox-1* and *fog-2 V; cox-1 M* G0 males transferred more sperm on average (191.5 and 245.1, respectively), than N2 males (120.7) (Dunnett’s test, *d* = 2.34, *α* = 0.05). Analysis of all lines showed a significant impact of the sexual system (*F*_1_ = 5.600, *p* = 0.037) on average sperm number, such that *fog-2 V; cox-1 M* lines transferred more sperm on average than *cox-1* lines, but no significant effect of treatment nor its interaction with the sexual system. For average sperm size, there was a significant impact of the interaction of sexual system with treatment (*F*_1_ = 7.676, *p* = 0.019) further described below, but not of either trait in isolation. We note that our measurements of sperm cross-sectional area are smaller than those previously reported for *C. elegans* N2 (e.g., Gimond et al., 2019), but fall within the normal range for the species (e.g., Murray et al., 2011). Our method differs from those of other studies, however, which typically measure the size of spermatids released from dissected male gonads into sperm medium on Petri plates.

The G60 *cox-1* RC line B3 was omitted from the analyses due to a lack of males. Among the seven other RC lines evolved from *cox-1* G0, all but one (B6) had evolved consistently larger sperm size by G60 (Dunnett’s tests, *p* < 0.05; an average of 1.98 um^2^ compared to 1.61 um^2^ for N2 G0), and all but one (again, B6) exhibited no significant change in average sperm number by G0. *cox-1* RC line B6 exhibited lower sperm number and no change in sperm size compared to *cox-1* G0 (Supplementary Table S2). By comparison, two-sided Student’s *t*-tests revealed that *fog-2 V; cox-1M* RC lines (as a group) had reduced sperm size (*t* = −2.406, *p* < 0.05) and number transferred (*t* = −3.418, *p* < 0.01) compared to their G0 strain, but no individual RC line was significantly different from the G0 control for either trait (Dunnett’s tests; *α* = 0.05). Average sperm size and number showed a non-significant positive correlation in *cox-1* G0 lines (*ρ* = 0.588, *p* = 0.074); this relationship became significant across *cox-1* G60 RC lines (*ρ* = 0.338, *p* = 0.015). By contrast, there was no relationship between these traits for *fog-2 V; cox-1 M* G0, *fog-2 V; cox-1 M* G60 RC lines, or the N2 G0 control.

## Discussion

We aimed to provide an initial test of the mitonuclear sex hypothesis alongside an evaluation of biological changes associated with altered male frequency and outcrossing rates in one experimentally-evolved set of *C. elegans* nematodes experiencing mitonuclear mismatch with different mating systems. In partial agreement with this hypothesis, we observed the evolution of at least temporarily increased male frequency in at least one replicate line of all facultatively-outcrossing ETC mutant strains experiencing competitive laboratory conditions. These mutants included the nuclear *isp-1*, mitochondrial *cox-1* and *ctb-1*, and *isp-1 IV; ctb-1 M* double mutant as reported here, and the nuclear *gas-1* as previously reported (Wernick et al., 2019). By contrast, evolved N2 control lines exhibited no change in male frequency across the 60-generation experiment (Figure 1; Supplementary Tables S1, S2) as in our earlier studies (Estes and Lynch, 2003; Denver et al., 2010; Katju et al., 2015). This may suggest that the mitonuclear mismatch experienced by the ETC mutant lineages presented a selective challenge beyond that imposed by food scarcity, and that this challenge may have favored the presence of greater-than-wildtype frequencies of males and outcrossing. There were ETC strain-specific differences in the timing of high-male frequency origin, the duration of elevated male frequency, and the number of replicate RC lines affected, but there was no tendency for high male frequency to be more strongly associated with either mitochondrial or nuclear ETC mutant alleles. Although male frequency was not tracked across generations in our previous experiment with *gas-1*, we noted that they had reached approximately 50% for affected lines by midway through the 60-generation experiment (Wernick et al., 2019), similar to the pattern observed here for the *cox-1* RC lines (Figure 2). Because we found no evidence for elevated rates of X-chromosome nondisjunction, we can exclude the possibility that parallel mutations in sex-determining genes (Table 1 in Anderson et al., 2010) caused the higher male frequencies in our study. In fact, we suspect that the parallel changes in male frequency in these lines are unlikely to be genetic in nature (we found no evidence for this in our previous genomic analysis of evolved *gas-1* lines; Wernick et al., 2019). Rather, we propose that males, which were arising in the facultatively outcrossing lines at wildtype frequencies from the outset of the experiment (e.g., Figure 2), and associated outcrossing were favored in ETC mutant RC lines owing to features of their genetic and/or population genetic environment; e.g., mitonuclear mismatch coupled with strong selection. However, we are not ruling out a role for genetic (or epigenetic) changes as there is precedent for rapid parallel and convergent adaptation driven by *de novo* mutation in *C. elegans* laboratory populations (Denver et al., 2010; Wernick et al., 2019).

Although we evaluated outcrossing rates for only two *cox-1* line sets (*cox-1* and *fog-2 V; cox-1 M*) at only two generational timepoints (G0 and G60), our results suggest that the presence of males did in fact translate into some degree of outcrossing during our study—perhaps at least 36% for facultatively outcrossing *cox-1* lines and the expected 100% for obligately outcrossing *fog-2 V; cox-1 M* lines based on G60 values. We emphasize that outcrossing rates as typically measured for *C. elegans* reflect only the ability of experimental pairs (or groups) to outcross, not the actual level of outcrossing that was occurring at any particular generational time point, which would depend on the frequency of males at that time point. This is especially true for the N2 G0 strains and its G60 RC lines, and the *cox-1* G0 strain, all of which exhibited very low (wildtype) male frequencies. For these lines, the actual rates of outcrossing would have been similarly low.

The early temporal patterns of male frequency in evolving *cox-1* RC lines, characterized by an initial increase followed by a decline (Figure 2), were similar to patterns of outcrossing from previous studies of wildtype lines exposed to increased mutation and selection pressure (e.g., Figure 1D in Morran et al., 2009b). This is notable since our study and that of Wernick et al. (2019) began with inbred isogenic strains rather than with genetically variable populations as in all previous *C. elegans* studies that documented elevated male frequency and outcrossing (reviewed in Cutter et al., 2019). There would clearly need to be sufficient genetic variation arising during the experimental evolution phase for males and outcrossing to have been favored over selfing, as we have previously documented (e.g., Denver et al., 2010; Wernick et al., 2019). Although estimated per-generation rates of mtDNA mutation are not especially high (Konrad et al., 2017), the per-genome rates could be vastly underestimated owing to selection within individuals (i.e., among mtDNA genomes or organelles) that cannot be avoided in standard mutation-accumulation experimental designs (Schaack et al., 2020). Furthermore, both gene copy number and mtDNA genome copy number changes could have contributed to such variation. Whether sufficient levels of either mitochondrial or nuclear mutational variance were introduced into *cox-1* lineages to render sexual outcrossing at least temporarily beneficial requires genomic analysis, but if this is the case, outcrossing would have increased the rate at which new mitochondrial-nuclear genome combinations were generated and became available for screening by selection as compared to selfing. Unlike previous studies, a resurgence of male proportion occurred in all but one of the *cox-1* RC lines by generation 70–80 (Figure 2). We speculate that the undulating pattern of male frequency in the remaining *cox-1* RC lines was a consequence of populations first achieving (or nearly achieving) an optimal multi-locus genotype *via* outcrossing-mediated adaptation—a time during which males and outcrossing would be initially favored, and then disfavored once the optimum was reached (Whitlock et al., 2016)—then losing the optimal genotype through residual outcrossing that would continue to occur until male frequency returned to the near-zero wildtype state. At this point, males and outcrossing might once again be favored and the cycle would repeat. Under the scenario, levels of population fitness would also fluctuate, lagging behind those of male frequency. This is supported by the fact that G60 *cox-1* RC lines tended have higher fitness (Figure 4) and lower male frequency (Figure 2) compared to the case at G80-87, at which point fitness was lower and male frequency higher. Whether this oscillating pattern of male frequency and fitness would have continued is of course unknown, but future WGS analysis of the *cox-1* RC lines at different generational timepoints will allow us to directly test the ability of outcrossing to reduce mitonuclear incompatibility, and for compensatory mutation to lessen the impact of the *cox-1* mutation.

Our *cox-1* mutant strain exhibited only slightly reduced fitness relative to the N2 wildtype (ns; Figure 3). This may seem incongruous with our previous statement that mitonuclear mismatch in this strain was exerting a selective force; however, the substitution is known to be associated with deleterious physiological traits such as elevated mitochondrial matrix oxidant burden and reduced oxidative stress resistance (Dingley et al., 2014). The authors also found that *cox-1* animals had elevated complex IV activity, which suggests a degree of physiological compensation for the mutation, as documented for other mitochondrial ETC mutants (Falk et al., 2008). Thus, while fitness as we have measured it—in a benign laboratory environment with ample food resource—may be fairly robust to these negative physiological phenotypes, there was likely room for genetic improvement within *cox-1* populations evolving under competitive conditions. We previously found that measures of competitive fitness were positively correlated with those made in benign environments, but with competitive fitness being the less sensitive of the two measures by far (Figure 3 in Wernick et al., 2019). We note that our result differs from that of that of Zhu et al. (2019) who found reduced progeny production for a similar *cox-1* cybrid strain generated from the CB4856 natural isolate relative to N2. Their assays began with L4-stage animals rather than embryos and utilized a different N2 background that was more fecund that ours. The difference in results is perhaps unsurprising given the potential for genetic variation among N2 strains from different laboratories (Sterken et al., 2015) and for a large impact of mitochondrial-nuclear epistatic effects on fecundity, known from studies of Caenorhabditid recombinant inbred lines (Chang et al., 2016; Zhu et al., 2019).

Our previous experiment with *gas-1* mutant *C. elegans* found a strong tendency for replicate lines that evolved high male frequency to also achieve greater fitness recovery during laboratory adaptation, suggesting that increased outcrossing was beneficial for adaptation (Wernick et al., 2019). Together with findings from the current study, we might tentatively conclude that males and sexual outcrossing are most beneficial to populations evolving from a state of low ancestral fitness (*gas-1*) and less beneficial or deleterious to those evolving from a state of higher ancestral fitness (*cox-1*). This is to be expected as the supply of potentially beneficial allelic combinations that could be generated *via* outcrossing would decline as populations near an adaptive peak or plateau (Fisher 1930; Martin and Lenormand, 2008). Consistent with this idea, the obligately outcrossing *fog-2 V; cox-1 M* lines exhibited no change in fitness—interestingly, this occurred alongside diminished outcrossing and male function, which is consistent with female-specific components of fitness having experienced stable or upward evolution over the same timescale (Table 2)—while the facultatively outcrossing lines showed slight upward evolution of self-crossed but not of outcrossed fitness, and all but one of the obligately selfing *xol-1 X; cox-1 M* lines evolved increased fitness (Figure 3B, Table 2). However, the amount of adaptation achieved by the obligately outcrossing *fog-2 V; cox-1 M* RC lines was likely influenced by synergistic epistasis between its two mutant alleles (Figure 3). Specifically, the *fog-2 V; cox-1 M* G0 ancestor began the experiment with higher fitness than the *fog-2* single-mutant strain, indicating a beneficial effect of *cox-1*-bearing mitochondria against a *fog-2* nuclear background. The minimal evolutionary change experienced by *fog-2 V; cox-1 M* RC lines may therefore be a result of its G0 ancestor beginning the experiment closer to an adaptive peak than either *cox-1* or *xol-1 X; cox-1 M*. Future analyses of fitness data currently being collected from the complete set of ETC mutant lineages (with variable starting fitnesses) generated by this study will provide more insight into this question.

**TABLE 2:** Direction of trait evolution in G60 *cox-1* RC lines compared to their G0 mutant ancestor. The fitness of the facultatively outcrossing *cox-1* RC lines was assayed by self-crossing (s) and outcrossing (o) relative to the selfed or outcrossed G0 ancestor, respectively.

	*ω*	Outcrossing rate	Male sperm number	Male sperm size	Male mating success with coevolved	Male mating success with tester
*xol-1 X; cox-1 M*	+					
*cox-1*	+ ^s^					
*cox-1*	= ^o^	−	=	+	−	+
*fog-2 V; cox-1 M*	=	−	−	−	−	−

The difference in relative fitnesses of G60 *cox-1* RC lines evaluated *via* selfing versus outcrossing (Figure 3) suggests that hermaphrodites’ self-crossed fitness, a trait previously found to be positively correlated with population-wide fitness in *C. elegans* (Carvahlo et al., 2014), increased more than did their outcrossed fitness during the experiment. Meanwhile, evolution of high male frequency in the same lines was accompanied by increased male sperm size and mating success with tester females, compatible with previous studies showing that increased male-male competition leads to evolution of traits that benefit males (e.g., Palopoli et al., 2015) and suggesting that evolution of sperm morphology was not hampered by poor-functioning mitochondria in *cox-1* lines. However, increased male frequency in *cox-1* RC lines was also accompanied by reduced mating success with coevolved hermaphrodites and slightly reduced outcrossing ability compared to G0 males. Taken together, these results may be indicative of intersexual conflict, perhaps mediated by prezygotic effects; e.g., increased competitiveness of hermaphrodite sperm or expulsion of male sperm (Kleeman and Basolo, 2007). Of the two *cox-1* RC lines (B6 and B7) where hermaphrodites evolved significantly higher selfed fitness than the G0 ancestor, both had unchanged fitness when assayed by outcrossing. Males from line B6 had significantly smaller and fewer sperm than other RC lines (Supplementary Table S2). Future work could explore whether line B6 hermaphrodites evolved larger or more sperm during the experiment, pointing to sexually antagonistic coevolution, although no correlation between Caenorhabditid male and hermaphrodite sperm size has yet been discovered (Gimond et al., 2019).

The obligately selfing *xol-1 X; cox-1 M* G60 RC lines achieved large fitness gains compared to their G0 ancestor, far beyond those of all other strains (Figure 3). These fitness gains were due to a substantial increase in early-life reproduction, which has an outsized influence on *ω*, as well as to increased lifetime reproduction (Supplementary Table S2). The G60 *xol-1 X; cox-1 M* RC lines produced an average of 102 surviving offspring across the first 2 days of reproduction, 46 more than their G0 ancestor and 30 more than the N2 wildtype. By contrast, their average lifetime productivity was 256, which was 53 more offspring than their G0 ancestor and 43 more than N2. Because N2 is maximally laboratory-adapted, the opportunity for new beneficial mutations should be minimal. The *xol-1 X; cox-1 M* G0 strain began the experiment with slightly lower fitness than N2 (ns), but as the fitness of its G60 RC lines exceeded that of both the G0 and wildtype N2, it may be that mitonuclear mismatch alongside evolution under selfing unlocked new areas of the adaptive landscape (cf., Burch and Chao, 2000), the shape of which will depend on the nature and extent of, in this case, *mitonuclear* epistasis (c.f., Weinreich et al., 2005). Although effective recombination between mitochondrial and nuclear genomes will be much reduced for *C. elegans* reproducing by selfing versus outcrossing, it will still occur. This minimal level of intergenomic recombination may have been sufficient to generate new beneficial mitonuclear genotypes in *xol-1 X; cox-1 M* lines, but also sufficiently low to avoid breaking up these combinations in subsequent generations. In this case, selfing could quickly drive such genotypes to fixation in the population. The benefits of selfing versus outcrossing will also depend on the degree of dominance of beneficial mutations. Because some of these mutations may be recessive (Joseph et al., 2014), benefits of selfing in our system may have extended both from preservation of coadapted allele combinations, and from realizing the full advantage of recessive beneficial mutations. We note that our study shares the same weakness as that of Morran et al. (2009b) in that effective population size (*N*_*e*_) is different between lineages evolving as selfing or outcrossing. However, the lower *N*_*e*_ expected under selfing, and the absence of sexual selection, apparently did not prevent the fixation of beneficial mutations in the *xol-1 X; cox-1 M* RC lines.

We provide the first controlled study of the influence of different forms of mitonuclear mismatch on the evolution of mating system dynamics and fitness. We also provide an imperfect test of one tenet of the mitonuclear sex hypothesis (Havird et al., 2015)—imperfect because the hypothesis specifically predicts an advantage of outcrossing over asexuality rather than of outcrossing over self-fertilization. With that caveat, we observed an increase in male frequency (and presumably, outcrossing) in at least one replicate of all facultatively outcrossing lines in accordance with this hypothesis, alongside increased male sperm size and mating success in affected *cox-1* lines. However, more work is required to understand the exact mechanism by which male frequencies were increased, how such increases relate to hermaphrodite individual fitness, and the implications of this study for mitochondria-centered hypotheses for the evolution and maintenance of sex. Despite the observation of increased male frequency in many facultatively outcrossing RC lines, obligate self-fertilization did not hinder fitness evolution in mitochondrial *cox-1* mutant RC lines. Comparing results to those of our previous study (Wernick et al., 2019) suggests that the benefit of outcrossing to an evolving population may depend on its level of ancestral fitness, although we are as yet unable to compare evolutionary trajectories of mitochondrial and nuclear ETC mutants of similar starting fitnesses. This is difficult as mitochondrial ETC mutations that are homoplasmic (fixed) tend to have minor fitness impacts relative to nuclear ETC mutations; mtDNA mutations exceeding 60–80% heteroplasmy are rarely observed (but see Konrad et al., 2017 and Dubie et al., 2020) and assumed to be lethal at the organelle, cell, or tissue level (Rossignol et al., 2003). Our design would also have benefited from including non-ETC mutant ancestors to understand whether the impacts of mitonuclear mutations on the evolution of mating system dynamics and fitness are unique or similar to those of other deleterious mutations. However, a previous study that documented laboratory adaptation in a large set of *C. elegans* mutation-accumulation (MA) lines, which would have contained a variety of mutation types, found no evidence of elevated male frequency; rather 1 MA line with a high incidence of males (Him) phenotype reverted to a wildtype frequency of males after 60 generations of evolution (Estes and Lynch, 2003). Future phenotypic and whole-genome analyses will test hypotheses about the genomic locations and functional nature of compensatory mutations, and directly test the effect of reproductive mode on mitonuclear evolutionary dynamics.

## Data Availability

The datasets presented in this study can be found in online repositories. The names of the repository/repositories and accession number(s) can be found in the article/Supplementary Material.
