# Supplementary figures and images for: Mitonuclear Mismatch is Associated With Increased Male Frequency, Outcrossing, and Male Sperm Size in Experimentally-Evolved C. elegans
Source: Front Genet. 2022 Mar 11;13:742272. doi: 10.3389/fgene.2022.742272 (PMC8961728; doi:10.3389/fgene.2022.742272)

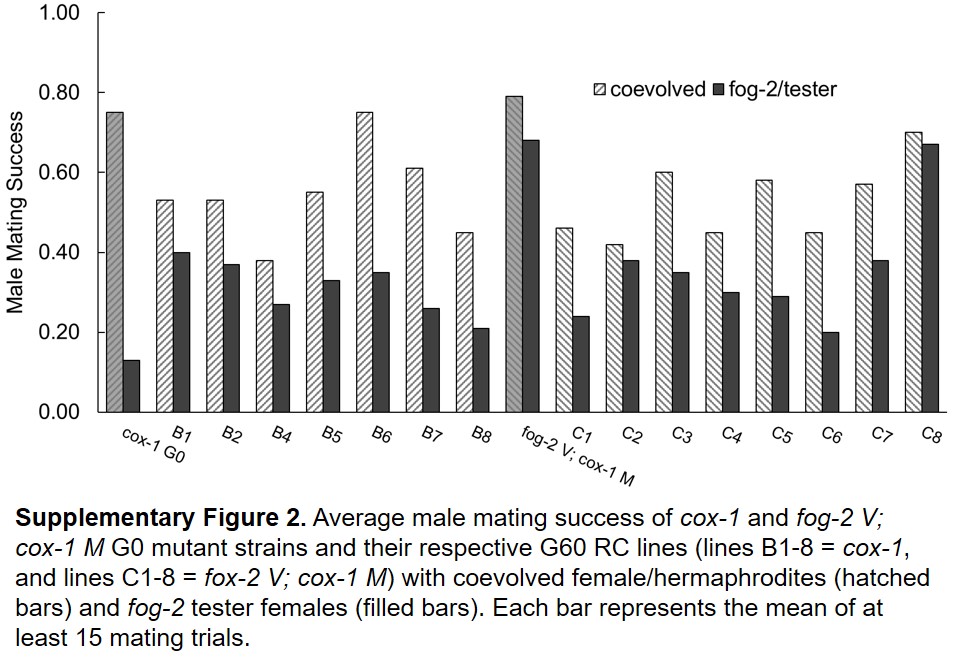

Supplement: Supplementary file 2 [file Image2.jpg]

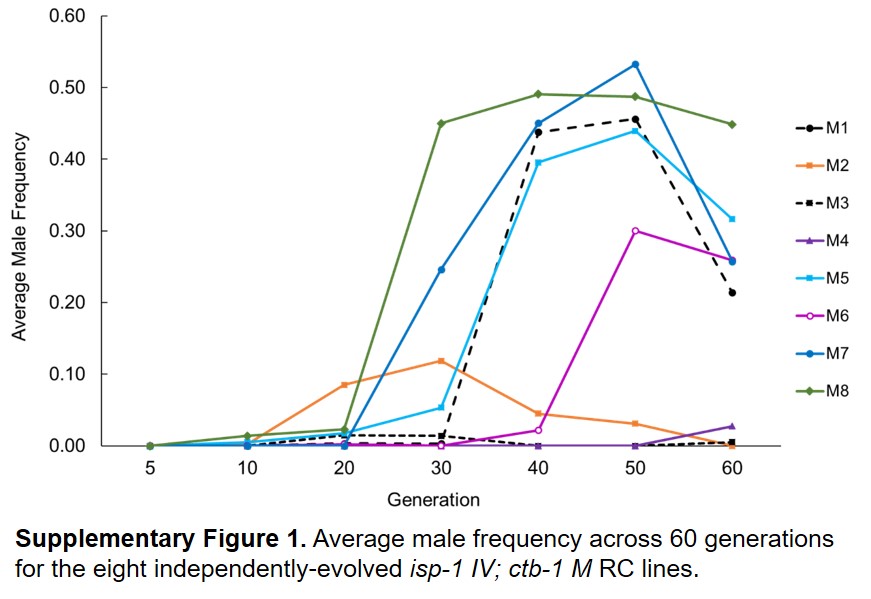

Supplement: Supplementary file 4 [file Image1.jpg]
